# Risk factors of induced abortion among preparatory school student in Guraghe zone, Southern region, Ethiopia: a cross-sectional study

**DOI:** 10.1186/s12905-019-0813-3

**Published:** 2019-09-11

**Authors:** Kifle Lentiro, Teklemichael Gebru, Abdusemed Worku, Agizie Asfaw, Tigist Gebremariam, Addisu Tesfaye

**Affiliations:** 1 Department of Public Health, Medicine and Health Science College, Wolkite University, Wolkite, Ethiopia; 2 Department of Medicine, Medicine and Health Science College, Wolkite University, Wolkite, Ethiopia

**Keywords:** Abortion, Risk factors, Preparatory school female, Guraghe zone

## Abstract

**Background:**

Induced abortion is a common undergo in many societies of the world. Every year, around 20 million unsafe abortions are done worldwide. From fragmented studies conducted in Ethiopia, the prevalence of induced abortion and its adverse effects are increasing over time. The aim of this study was to assess factors associated with induced abortion among female preparatory school students in Guraghe zone.

**Methods:**

A cross-sectional study was conducted among female students of preparatory schools in April 2017. Systematic random sampling technique was employed to select 404 students from the total of 3960 female preparatory school students in the study area. Data was collected through self-administered questionnaires. Descriptive summary, binary and multivariate analyses were underwent to identify factors associated with induced abortion. The study was ethically approved by institutional review board of Wolkite University.

**Results:**

The response rate of this study was 98.3%. The lifetime prevalence of induced abortion among young preparatory schools students whose age range from 15 to 22 years was 13.6% [95% CI (10.4, 17.1)]. The odds of induced abortion undergo was 2.3 times more likely in rural family residents [AOR = 2.3, 95% CI (1.1, 4.8)] as compared to that of urban family residents. Students without sexual health education were 6.4 times more likely to undergo induced abortion as compared to those who got sexual health education at sc0000hool [AOR = 6.4, 95% CI (3.1, 13.1)]. Furthermore, students who drank alcohol often were 4 times [AOR = 4.0, 95% CI (1.1, 14.2)] more likely to undergo induced abortion and students who consumed alcohol sometimes had 3.3 times [AOR: 3.3, 95%CI (1.4, 8.1)] the risk of induced abortion compared with girls with no history of alcohol consumption.

**Conclusion:**

A high lifetime prevalence of induced abortion among young adolescent was observed. Being rural residence, not having reproductive health education, and alcohol consumption were found to be independent predictors of induced abortion undergo. Therefore, IEC/BCC programs with special emphasis on youth friendly sexual and reproductive health services should be strengthened to reduce induced abortion.

## Background

Induced abortion is defined as termination or initiation to terminate pregnancy before 28 weeks of gestation or less than 1000 g fetal weight intentionally. In certain practical circumstance; it may be deemed as safe or unsafe [1, 2]. The World Health Organization (WHO) estimates that every year, nearly 5.5 million African women undergos unsafe abortion. More than 36,000 of these women die from complications of the procedure, whereas millions more experience acute or chronic illness that may lead to disability. In developing countries half of all maternal death is estimated to be due to unsafe abortion, with as much as 14% of the deaths occurring in sub-Saharan Africa [3]. Acute complications of induced abortion include; infection, cervical and uterine trauma and haemorrhage. Long-term post-abortion complications include secondary infertility. Besides the short and long-term complications, adolescents’ also suffer with emotional problems which may be due to social stigma [4].

In Ethiopia, the demand for induced abortion is common in the rural community, and may be associated with low contraceptive use and high levels of unwanted pregnancy. For instance, only 32.4% of Ethiopian rural women of reproductive age use modern contraceptive and more than 40% of pregnancies are unplanned. In 2008, an estimated 382,000 induced abortions were reported, and 52,600 women were suspected to have post-abortion complications [3–5].

According to the Ethiopian Demographic and Health survey (EDHS) 2016, the Maternal Mortality Ratio (MMR) was estimated to be 412 per 100,000 live births. This ranks the country as having the fifth largest number of maternal death [3, 6, 7]. According to Ethiopian Ministry of Health (MOH) 2010 report, 32% of all maternal deaths in Ethiopia was related to unsafe abortion [8].

In effect, the Ethiopia government revised the laws of abortion in 2005 that had permitted induced abortion service in restricted situations such as: if woman’s pregnancy could create health problems on her, if the foetus had conditions incompatible with life, or if the conceived pregnancies were from incest, rape or minor groups of youngsters [7, 9].

### Statement of the problem

Of 210 million pregnancies that occur in each year, about 46 million (22%) end up being aborted. Approximately 20 million (43%) of those abortions are probably underwent by someone without having the skills or understanding the procedure in an ideal health facility, or both [3]. Every year, more than 70, 000 women die as a result of unsafe abortion and hundreds of thousands may eventually suffer from a serious health consequence, and often, a permanent disability [1]. According to Centre for Disease Control and Prevention (CDC) report from the United State of America (USA) induced abortion among adolescents aged 15–19 years accounts 14.6% of all abortions or 12 abortions per 1000 adolescents [10].

A nationwide study in Ethiopia 2008 indicated that an estimated 382,000 induced abortions were underwent and 52,600 women were treated for complications of abortions. There were an estimated 103,000 legal abortion procedures underwent in health facilities of the country. From different studies and report, the burden of induced abortion and its negative consequences keep increasing over time in the country [11].

Furthermore, the likelihood of short- and long-term complications among abortion-undergod mothers were 20 times higher than her non abortion-undergod counterpart [12]. Being adolescent is a phase for lifestyle and behavioral changes. In addition, at this age students are living away from their parents for the first time. Because of inaccessibility of nearby schools, many are forced to re-locate to distant towns, and to live in rental accommodation without parental supervision. This may increase the risks of unsafe sexual exposure and involvement, leading to un-intended pregnancy. In essence, at this age may often a unique setting to study the possible contributory factors which lead to unsafe sexual behavior, un-intended pregnancy, induced abortion and its various consequences.

In Ethiopia and to the best of our knowledge, there is no published article focused on induced abortion among secondary school students. Therefore, the aim of the study was to assess the magnitude and predisposing factors of induced abortion among Guraghe zone preparatory students. We are of the opinion that this study may help offer insights that could contribute to designing an effective intervention strategy in Ethiopia and beyond.

## Methods

### Study area and period

The study was conducted in Guraghe zone preparatory schools between April 1 to 30/2017. Guraghe zone is located in South Ethiopia. In this zone there were 31 public secondary schools during data collection period of which 12 were preparatory schools that had 7141 students on their roll. Of which 3960 of them were female students [12].

### Study design and population

A cross sectional study design was employed to assess the magnitude and associated factors of induced abortion among female preparatory students in Guraghe zone. All female students of Guraghe zone preparatory schools were source population whereas randomly selected female students of Guraghe zone preparatory schools were study population.

### Sample size determination and procedure

The required sample size for the study was calculated using Epi-Info 7 Stat Calc for window by assuming; 22% prevalence of abortion in the region [13], 95% confidence interval, 4% margin of error and 90% expected response rate. Accordingly, the calculated sample size for finite population was 411 female students. After preparing a sampling frame, systematic random sampling technique was employed to select the study units. Sampling interval was calculated by dividing total cumulative population (3960) into the calculated sample size, giving approximately: 10. Using the Microsoft Excel random number generating tool, numbers between 1 and 10; 4 were randomly selected. The 4th female from the list was the first sample and the second sample was the 14th order of the cumulative frequency and the rest samples were identified in the same fashion.

### Data collection and quality assurance

Data was collected using adapted self-administered questionnaire that consists socio-demographic characteristics (8 item), knowledge related (8 item), accesses to service (4 item), contraceptive use (3 item), history of induced abortion (one item), reasons to abortion (3 item), and consequences of abortion (2 item) with yes/no or multiple choice responses. The questionnaire was adapted in English and translated into the local language (Amharic) and then retranslated back into English by another reasonably-skilled translator. Supervision and daily based check-up on the field was made by the research team.

The data collection tool was pre-tested on 5% of the calculated sample size. Three days of training was given to school unit leaders prior to the process of data collection and the need to assure confidentiality for all respondents. Furthermore, double data entry (protection) was made using Epi-data software for validation.

### Data processing and analysis

Data processing and analysis was made by using Epi-data 3·1 and SPSS version 23·0 statistical software for window, respectively. A descriptive statistical summary like mean and proportions were computed. To avoid unstable estimate, independent variables with *p*-value ≤0·25 found in the first binary screening analysis were further considered into the final model [14]. Backward stepwise logistic regression was applied to describe the functional relationship between independent factors and the outcome variable. A point estimate of Odds Ratio (OR) with 95% confidence interval (CI) was computed to estimate the strength of association between independent and dependent variable, induced abortion. For all statistical significant tests, *p*-value < 0·05 was used as a cut-off point.

## Results

### Socio-demographic characteristics

A response was obtained from 404 female respondents, giving the response rate of 98.3%. The mean age of study participants were 17 years with a standard deviation of one. More than half of the study participants were Orthodox faith: 248 (61.4%) followed by Muslim: 108 (26.8%) by religion. Around two third of the respondents’ parents were from rural residence: 266 (65.8%). The lifetime prevalence of induced abortion among respondents was: 55(13.61%), with 95%CI (10.4 to 17.1%) (Table 1).

**Table 1:** Socio-Demographic Characteristics of the Respondents among Guraghe Zone Preparatory School Student, *n* = 404, April 2017

Variables	Induced Abortion		Chi-square	*P*-value
	Yes	No		
	Count (%)	Count (%)		
Age of respondent				
< 17	28(14.4)	167(85.6)	0.178	0.673
> =18	27(12.9)	182(87.1)		
Respondent’s education				
Grade 11	37(16.7)	185(83.3)	3.905	0.048
Grade 12	18(9.9)	164(90.1)		
Respondent’s religion				
Orthodox	31(12.5)	217(87.5)	2.746	0.432
Muslim	16(14.8)	92(85.2)		
Protestant	7(15.2)	39(84.5)		
Catholic	1(50.0)	1(50.0)		
Parents residence				
Urban	12(8.7)	126(91.3)	4.311	0.038
Rural	43(16.2)	223(83.8)		
To whom you live with				
Without family	21(16.8)	104(83.2)	5.324	0.005
With family	34(12.2)	245(87.8)		
Family education				
Not write and read	20(18.7)	87(81.3)	3.251	0.065
Write, read and above	35(11.8)	262(88.2)		
Monthly income				
< 500	22(13.8)	137(86.2)	0.38	0.998
501–700	3(13.0)	20(87.0)		
700–1000	9(14.1)	55(85.9)		
> 1000	21(18.7)	137(86.7)		

### Behavioural and knowledge factors

Exposure to sexual health education was admitted by 225 (55.7%) respondents. Among those who had no admission for sexual health education, 43 (24.0%) of them underwent induced abortion. Majority of the respondents, 221 (54.7%) did not support provision of induced abortion procedure for youngsters; whilst 310 (76.7%) believe that induced abortion has a risk on women’s health. More than three fourth of the respondents, 311(77%) who never consumed alcohol had never underwent induced abortion. However, among those who consumed alcohol, 40(9.9%) admitted to do so (Table 2).

**Table 2:** Behavioural and Knowledge Related Factors of Respondents among Guraghe Zone Preparatory School Student, *n* = 404, April 2017

Variables	Induced Abortion		Chi-square	*P*-value
	Yes	No		
	Count (%)	Count (%)		
Sexual Health education				
Yes	12(5.3)	213(94.7)	29.607	0.000
No	43(24.0)	136(76.0)		
Agreement on abortion				
Yes	7(31.8)	15(68.2)	8.481	0.075
Never	32(14.5)	189(85.5)		
Depends	12(10.4)	103(89.6)		
Sometimes	4(9.3)	39(90.77)		
Not sure	0(0.0)	3(100.0)		
Risks of abortion				
Yes	44(12.4)	310(87.6)	3.412	0.065
No	11(22.0)	39(78.0)		
History of alcohol consumption				
Yes often(daily/weekly 2-3x)	5(33.3)	10(66.7)	11.641	0.003
Yes sometimes (monthly 1-4x)	10(26.3)	28(73.7)		
No never	40(11.4)	311(88.6)		
Enforcing to abortion				
My morals	19(15.4)	104(84.6)	9.311	0.097
My religion	11(7.7)	132(92.3)		
The media	6(27.3)	16(72.7)		
Not dare	13(16.5)	66(83.5)		
Peers	5(18.5)	22(81.5)		
Others	1(10.0)	9(90.0)		
abortion is allowed				
If A woman that has been raped	20(16.4)	102(83.6)	9.001	0.061
If A women that will die if she does	16(9.7)	149(90.3)		
If A woman that is having an affair	4(40.0)	6(60.0)		
If A woman cannot have baby	6(12.8)	41(87.2)		
I don’t know	9(15.0)	51(85.0)		

### Reproductive health factors

Among the respondents who undergod induced abortion, 55 (13.61%) replied that the reason for their pregnancy was rape which accounts 10(18.2%). On the other hand, the main reason for abortion service demand was not to interrupt their on-going education 33(60.0%), followed by refusal of the pregnancy by sexual partner and fear of family and society in which both accounts 6(10.9%) (Table 3).

**Table 3:** Pregnancy and Abortion Factors of the Respondents among Guraghe Zone Preparatory School Student, *n* = 55, April 2017

Variables	Count (%)
Abortion frequency	
One	39(70.9)
Two	15(27.3)
Three	1(1.8)
Reasons for abortion	
Not to disrupt education	33(60.0)
Too young to bear a child	9(16.4)
Could not afford to cater for a	1(1.8)
Partner refused to accept pregnancy	6 (10.9)
Fear family and society	6(10.9)
Reason to pregnancy	
I am raped (violence)	10(18.2)
Unplanned Pregnancy	27(49.1)
Unprotected sexual intercourse	13(23.6)
Contraception failure	5(9.1)
Type of abortion procedure	
Safe	42(76.4)
Unsafe	13(23.6)
Does abortion has Complication	
Yes	22(40.0)
No	33(60.0)
Type of complication	
Excessive bleeding	14(60.9)
Pain	7(30.4)
Uterine perforation	1(4.3)

### Predictors of abortion

Both bivariate and multivariate analysis of the exposure variables were employed to identify the final predictors of induced abortion among preparatory school students. In bivariate analysis we revealed that; parent residence, respondent’s education, family education, sexual health education, agreement on abortion as safe, history of alcohol consumption and allowed abortion were significantly associated with induced abortion. After running the full multivariate logistic analysis; respondent’s educational level, family education, agreement on abortion as safe and allowed abortion were excluded (Table 4).

**Table 4:** Independent Predictors Associated with Abortion in Gurage Zone Preparatory School Students, n = 404, April 2017

Variables	Induced Abortion		OR with 95%CI	
	Yes	No		
	Count (%)	Count (%)	Crude	Adjusted
Parents residence				
Urban	12(3.0)	126(31.2)	1	1
Rural	43(10.6)	223(55.2)	2.03(1.03, 3.98)	**2.29(1.10, 4.77)**
Sexual health education				
Yes	12(3.0)	213(52.7)	1	1
No	43(10.6)	136(33.7)	5.61(2.86, 11.02)	**6.40(3.12, 13.11)**
History of alcohol				
No never	40(9.9)	311(77.0)	1	1
Yes often	5(1.2)	10(2.5)	3.89(1.27, 11.95)	**4.00(1.13, 14.22)**
Yes sometimes	10(2.5)	28(6.9)	2.78(2.26, 6.14)	**3.30(1.35, 8.06)**

Finally, female students from family of rural residents were 2.3 times more likely to undergo induced abortion as compared to those from urban residence [AOR: 2.3, 95% CI (1.10, 4.8)] with *p*-value of 0.04. On the other hand, young females with no sexual health education were 6.4 times more likely to undergo induced abortion than those who had sexual health education [AOR: 6.4, 95% CI (3.1, 13.1)] with a p-value < 0.00. Moreover, students who often consume alcohol were four times more likely to perform induced abortion, and those who consume alcohol sometimes were 3.30 times more likely to undergo it as compared to those with no history of alcohol consumption [AOR: 4.0, 95%CI (1.1, 14.2)] and [AOR: 3.3, 95% CI (1.4, 8.1)] with a *p*-values of < 0.01, respectively (Table 4).

## Discussion

In this assessment the lifetime prevalence of induced abortion was 13.6% which is consistent with a study done in Harare, Ethiopia which showed the prevalence of induced abortion was 14.4% [15]. However; a study done in Adwa high school (Northern Ethiopia) indicated that of 84.21% girls who had history of unintended pregnancy, 52.08% of these pregnancies were terminated by induced abortion [16]. Similarly another study done in Aleta Wondo (southern Ethiopia) high school students indicated that 15.3% had unwanted pregnancy, of these, 80% of them were terminated [17]. This might be due to the difference in socio-demographic characteristics of the respondents among southern and northern Ethiopia. Similarly a study done in Nigerian undergraduate students showed that 34% of all female respondents ever had an induced abortion [18]. These figures are also lower compared to those from developed countries: for example, in a 2015 report from the American college of paediatricians, up to 30.4% of USA teens who had un-intended pregnancy ended up with induced abortion [19].

In contrast to other studies which were done in Ethiopia; a 4.8% prevalence rate of induced abortion was seen in Northwest Ethiopia which is much lower than our study, implying that induced abortion is a hidden public health problem affecting women in reproductive age group in the study area. [20].

From this study we revealed that 18% of those with induced abortion reported pregnancy to be due to rape, this may hinder girl to get access for education and contraception. On the other hand, boys and men may need education to change social norms to respect girl’s/women’s bodily autonomy.

The major determinants of induced abortion in this assessment were parental residence, sexual health education, and alcohol consumption. Accordingly, female students whose family residences from rural were more likely to be exposed for induced abortion. Similar to our finding a study done in Aleta Wondo (Southern Ethiopia) showed that urban family residence was protective from premarital sexual exposure and its possible consequence of induced abortion [15]. This could be due to parental proximity and supervision or this might be due to lack of an open discussion about safe sexual health from the very beginning of adolescent age in the rural community.

On the other hand, young females with no sexual health education were more exposed to abortion than those who had sexual health education at school. Those who were not informed about sexual health were found to have a significantly higher chance of having induced abortion (AOR =2.8, 95% CI 1.4, 6.4) [21] and this possibly be because comprehensive sexual health information may impact on adolescences sexual life. Additionally, alcohol consumption was an important predictor as it is the conventional predisposing factor for sexuality in youths, so students who often consume alcohol were more prone to induced abortion than with no history of alcohol consumption because alcohol consumption obviously, exposed them for unprotected sexual intercourse. The earlier cited study from Wolita Sodo University and elsewhere revealed that alcohol use had statistically significant association with undergo of induced abortion [22, 23], and other study elsewhere found that students who consume alcohol had about four times more risk of experiencing induced abortion than students who never used alcohol [AOR = 3.95% CI(1.63–1.1)] [24].

In this study we acknowledged the following limitations. Most importantly, it lacks triangulation with qualitative findings to address unexpected issues, as well as it might be affected by a culture-based variation in self-disclosure and the information may be subjected to recall bias and social desirability bias. Furthermore, the study design does not allow establishing a cause-effect relationship.

## Conclusion

From this survey a remarkable high lifetime prevalence of induced abortion was observed among female preparatory students. Being parents’ rural residence, not getting sexual health education on abortion and being alcohol consumers were found to be significantly associated with induced abortion undergo. Thus, we recommended that; the Ethiopian Ministry of health and Ministry of education should work together with schools to design and execute Information, Education and Communications (IEC) programs emphasizing on sexual and reproductive health particularly on sex education, focusing on youth-friendly services, delaying sexual activity, access to contraceptive options and safe and legal abortion services to reduce un-intended pregnancy and induced abortion.

## Data Availability

The datasets used and analysed during the study available from the corresponding author on reasonable request.

## Abbreviations

AOR: Adjusted Odds Ratio
CDC: Centers for Disease Control and prevention
CI: Confidence Interval
EDHS: Ethiopian Demographic Health Survey
MMR: Maternal Mortality Ratio
MoH: Ministry of Health
OR: Odds Ratio
USA: United State of America
WHO: World Health Organization
